# Effects of a Nudging Cue Targeting Food Choice in a University Cafeteria: A Field Study

**DOI:** 10.3390/healthcare11091307

**Published:** 2023-05-03

**Authors:** Christine Kawa, Patrizia M. Ianiro-Dahm, Jan F. H. Nijhuis, Wim H. Gijselaers

**Affiliations:** 1 Department of Management Sciences, University of Applied Sciences Bonn-Rhein-Sieg, 53359 Rheinbach, Germany; patrizia.ianirodahm@h-brs.de; 2 Department of Educational Research and Development, School of Business and Economics, Maastricht University, 6211 LM Maastricht, The Netherlands; jfh.nijhuis@maastrichtuniversity.nl (J.F.H.N.); w.gijselaers@maastrichtuniversity.nl (W.H.G.)

**Keywords:** nudging, nudge acceptance, eating behavior, food choice, cafeteria setting, health intervention, students, immediate effects, field study

## Abstract

Many students approaching adulthood often choose high-calorie food products. Concurrently, health interventions applied during this life phase can potentially lead to a healthier lifestyle. Nudge health interventions in experimental cafeteria settings have been found to improve eating behavior effectively, yet research in real-world settings is lacking. Accepting nudges as health interventions impacts nudge effectiveness. The present study applies a pretest–posttest design for a period of three consecutive weeks (no nudge, nudge, no nudge), testing the effectiveness of the so-called Giacometti cue on the number of calories purchased in a real-world cafeteria. Students were exposed to the nudge during the intervention week when entering the cafeteria and when choosing their meals. After purchasing a meal, their choice was recorded, and they completed a questionnaire. The Giacometti cue immediately reduced the number of calories purchased (comparing weeks one and two). After nudge removal, an effect was identified, increasing the number of calories purchased (comparing weeks two and three). Contrary to expectations, higher nudge acceptance resulted in more calories purchased. Neither awareness of the nudge’s presence when buying food nor the interaction between acceptance and awareness played a role. We explore potential explanations for the Giacometti cue’s effects.

## 1. Introduction

An individual’s dietary behavior can be conceptualized as a food choice (behavior occurring before the food reaches the mouth, as in a food purchase), eating behavior (all outcomes related to actual food consumption, such as eating habits), and dietary intake or nutrition (all outcomes encompassing the content of the food consumed, such as caloric intake) [1]. Thus, any eating behavior and/or dietary behavior starts with a food choice that can have different caloric values. Diets involving calorie-dense foods high in sugar have been associated with several diseases, such as diabetes mellitus or an increased risk of heart disease [2].

A stage of life during which individuals are particularly prone to choosing foods high in calories is emerging adulthood during ages 18–25 [3,4]. During this life phase, many individuals participate in tertiary education as college or university students. The World Health Organization (WHO) describes the consumption of high-calorie foods as a risk to global health throughout the lifespan. Specifically, in highly industrialized countries, individuals do not consume enough fruit and vegetables and favor foods high in energy, fats, sugar, and calories [5]. For example, university students in Germany have been found to consume less fruit and vegetables than the amounts recommended by the WHO of five servings per day [6]. Less than 30% of Germany’s university students meet these WHO recommendations [7]. University settings are known for having a high availability of food products high in simple sugars and fats, such as ultra-processed foods that are dense in caloric value while lacking nutritional value [8]. At the same time, environmental factors, such as the living arrangements of students and whether they make food choices independent from their families, were found to play a role [8]. In addition, the environment in which a food choice is made has been found to influence this choice [9].

The Ottawa Charta describes the environment (setting) in which food is provided (for example, cafeterias) as relevant in implementing health interventions [10,11]. The WHO further suggests creating healthy food environments in which healthy diets are promoted [5]. Thus, applying health interventions within a cafeteria to foster a healthy environment can bring about positive outcomes for students, specifically because the majority of university students eat regularly in their university cafeteria [7,12]. Additionally, meal plans in cafeterias can be adjusted to contain more low-calorie food products in a cost-effective way [12,13]. Systematic reviews mention various promising strategies to improve dietary behaviors among university students (for example, cookery classes or nutrition labels on food products) [13,14]. These strategies have in common that the target group needs to be actively involved in activities requiring more of their time (e.g., joining food classes). Research shows that environmental cues can act as health primes that effortlessly lead individuals to reduce, for example, their consumption of high-calorie potato chips [15]. These environmental cues can yield favorable outcomes in complex, real-world settings in which individuals have limited cognitive decision-making capacities [15]. This is in line with the framework of situated interventions that describes situational cues embedded within the decision-making environment to change behavior effectively. This framework specifically involves environmental cues, such as nudges [16]. Taking this into account, interventions involving environmental cues may be more effective in achieving fewer choices of high-calorie foods.

There is a growing trend in research focusing on the effectiveness of environmental cues called nudges [17]. Nudges have demonstrated that they can be effective, unobtrusive interventions to influence food choices when students purchase food in cafeteria settings [12]. They circumvent any obstacles due, for example, to schematic cognitive biases, self-control, or procrastination problems [18]. Because everyday food choices are mostly made automatically and instinctively without long considerations, nudges are especially considered suitable for targeting this type of behavior [19,20].

A nudge is defined as “any aspect of the choice architecture that alters people’s behavior in a predictable way without forbidding any options or significantly changing their economic incentives” [18]. Thus, nudges can be small, subtle changes to the social and physical decision-making environment that steer the decision in a predictable direction [19]. The originators of the term nudging, Thaler and Sunstein, state that individuals often rely on heuristics or cognitive effects, especially when making fast decisions. Relying on these mental shortcuts often leads to suboptimal choices [18]. Nudges target these mental shortcuts by highlighting a specific choice within an environment [18]. Consequently, a nudge can be any form of environmental cue that steers behavior.

Many different types of nudges exist and can be classified into different categories. These categorizations often consider which heuristic, cognitive effect, or cognitive system is targeted [20,21,22]. Nudges are said to be based on the dual-process theory of the mind [23]. Many nudges function by activating an individual’s automatic decision-making processes (System 1) and therefore affect fast and intuitive decisions [24]. A nudge that activates automatic processes is, for example, a default setting in which a customer always receives a salad as a side dish instead of French fries. Nudges can also impact an individual’s deliberate and conscious decision-making processes (System 2) by activating reflective thinking processes [25]. Then, a nudge can, for example, provide specific information relevant to the given food choice scenario initiating conscious deliberation processes [25]. In summary, nudges influence behavior by making an optimal choice more explicit using simple environmental cues in a decision-making context. Therefore, nudges are a promising low-cost food choice intervention suitable in complex, real-world settings.

In general and across different research settings, the meta-analyses and literature review studies point out that food choice nudges can be considered to be effective, yielding moderate-to-high effects [19,20,26,27] of, for example, a Cohen’s *d* effect size of 0.43 [27]. However, this positive image of nudging fades away when taking into account whether the research findings on nudges were collected in lab settings or in real-world field settings. Research findings for real-world settings demonstrate rather mixed results, revealing weak or moderate effect sizes [19,21]. For example, food choice nudges tested in real-world field experiments show a Cohen’s *d* effect size of 0.23, which is equivalent to a reduced calorie consumption of 124 kcal per day [21]. In general, it appears that the effect sizes of nudges targeting dietary behavior are smaller in real-world field settings than in controlled laboratory settings. As a consequence, more research on nudging in real-world food choice contexts has been called for [19]. There is a limited understanding of which conditions enable the potential effects of nudges [28,29]. The present study addresses this call by focusing on how nudges target food choice behavior in a typical real-world field setting.

For the present study, a suitable environmental cue that primes eating behavior and reduces the consumption of high-calorie food products is the Giacometti cue [15,30]. It is based on the artwork of Alberto Giacometti and shows skinny, human-like sculptures [31]. The application of this environmental cue corresponds with the concept of nudging and is particularly suitable for implementation in the real world because it does not involve higher cognitive capacities to be effective [15]. Research shows that immediate exposure to Giacometti’s skinny sculptures can reduce the consumption of high-calorie chocolate with a Cohen’s *d* effect size ranging from 0.39–0.65 [32]. The underlying explanatory mechanisms of the Giacometti cue are described as priming weight-related mental concepts that lead, for example, to reduced consumption of high-calorie foods [30]. Priming effects primarily target the automatic decision-making processes of System 1 [23]. The Giacometti cue’s immediate effectiveness has been shown for individuals ranging from 35–39 years of age [30,32], and it was applied before in a real-world field setting in the form of a poster next to a vending machine. At that time, it was able to increase healthy snack choices made [33]. While it is immediately effective for older individuals, e.g., [32], it has not been tested on young adults. Moreover, it has only been tested in a real-world field setting involving snack purchases made from a vending machine [33] and dietary behavior at home [34] but not involving actual food choices made in a real-world university cafeteria. In the present study, we formulate several research questions focusing on the implementation of the Giacometti cue in a real-world university cafeteria targeting the dietary behavior of students on the threshold of adulthood. The first research question concerns the general and immediate effectiveness of this cue in this specific setting: (1) What is the immediate effect of the Giacometti cue on actual food purchases in a real-world university cafeteria?

The research so far on nudging has seldom ascertained the longitudinal effects of nudges or effects over time [35,36]. A systematic review on nudges targeting dietary behavior acknowledges that more research needs to be conducted assessing nudge effects at different points in time—for example, after the removal of a nudge or longitudinal effects [26]. A study assessing the effects of a nudge aiming to increase the sales of vegetarian dishes in a university cafeteria after it had been removed from the setting showed that nudging can lead to persistent changes over time [37]. Sales of vegetarian dishes were 6% higher in the intervention week (when the nudge was present) than in the baseline week (before the nudge was implemented). The nudge in this study made vegetarian dishes more visible, for example, by moving them into a more prominent position for the customer during the decision-making process [37]. Even in the posttest period (after the visibility nudge had been removed), sales of vegetarian dishes were 4% higher than in the baseline period [37]. It has been suggested that the long-term effects of nudges may depend on whether the nudge targets automatic (type 1 nudge) or deliberate cognitive processes (type 2 nudge) [38]. Type 1 nudges may elicit lasting effects by possibly creating new habits that override the immediate desire [36]. The visibility nudge described above qualifies as a type 1 nudge. Similarly, the Giacometti cue qualifies as a type 1 nudge targeting automatic cognitive processes [30]. In order to understand its longitudinal effects, one study assessed long-term exposure for six months to this cue. In this study, it was found to be effective in leading to weight loss after a six-month-long exposure to the nudge [34]. The nudge was presented on the material used in a weight loss program targeting individuals wishing to lose weight [34]. To the best of our knowledge, this is the only study involving any longitudinal effects of the Giacometti cue. That study assessed weight loss over time induced by the six-month-long exposure to the Giacometti cue. It did not assess what happens after the weight loss program (thus, the exposure to the Giacometti cue) ended [34]. The question of what happens when a nudge, and specifically the Giacometti cue, is removed is particularly interesting because, by definition, a nudge needs to be present within the decision-making context to have an effect [16,18]. In a lab setting, a randomized-untreated (no nudge) control group design with a pretest and posttest would be an obvious choice for assessing nudge effects because it controls for threats to internal validity. However, in a real-world setting such as a university cafeteria, it is necessary to consider that the cafeteria is visited by different visitors on different days. In such a situation, it requires us to deal with non-randomized samples and examine the effects of introducing and removing the treatment. This requires a research removed-treatment design with pretests and posttests, closely approximating meeting the research requirements for having a no-treatment control group [39,40]. In a field setting, it is essential to assess the effects of removing the Giacometti cue. Because it provides proxy estimates for the effects of introducing the cue, and effects after removing the cue, assuming that the respondent groups are comparable. In conclusion, when considering the first week of collecting data as a pretest (no Giacometti cue), the second research question asks: (2) How does the removal of the Giacometti cue from a real-world university cafeteria affect the actual food purchases?

A quantitative review of effect sizes concerning nudges concluded that nudge effectiveness relies in part on how it is perceived by the individual [41]. According to the Nudge Acceptance Model [42], nudge acceptance is linked directly to nudge effectiveness. Highly accepted nudges are more effective. The nudge technique, i.e., how the nudge is designed and used, also affects nudge effectiveness. Nudges have differential outcomes depending on which cognitive processes they tap into (automatic System 1 or reflective System 2 processes). This relationship between nudge technique and effectiveness is mediated by nudge acceptance [42]. Thus, nudge acceptance plays a crucial role in the successful implementation of a nudge [43]. While there is a consensus that nudges are generally accepted by the public at large, little is known about the conditions under which nudges are viewed as acceptable [44]. In addition, nudge acceptance has been found to vary from type of nudge to type of nudge [21,45,46] and also from target person to target person [22,47]. Specifically, university students seem to be more or less susceptible to nudges and accept nudges differently [48]. The relationship between nudge acceptance and nudge effectiveness has seldom been tested in empirical research in general and in real-world field studies specifically [19]. While all of this is known about nudging in general, little is known about the acceptance of the Giacometti cue as a health intervention and how this impacts its effectiveness. The research so far shows that the Giacometti cue induces a rather low acceptance rate in university students when it is assessed in a questionnaire without showing a picture of the cue [48]. So far, its acceptance has not been assessed in a real-world setting. When this nudge is applied in a real-world public setting (such as a cafeteria), it cannot be guaranteed that only a specific target group (for example, older individuals) is exposed to it. Researching the specific situation in which an intervention is implemented is always necessary [16]. Therefore, assessing the effects of the Giacometti cue, as well as its acceptance, are crucial. To first establish the relationship between the acceptance of the Giacometti cue and real-world food purchases, which is the dependent variable assessing nudge effectiveness in the present study, we formulate two additional research questions: (3) What is the role of acceptance of the Giacometti cue on actual food purchases in a real-world university cafeteria? (4) Does acceptance of the Giacometti cue moderate the nudge’s immediate effect on actual food purchases in a real-world university cafeteria?

In explaining nudge effectiveness, the growing body of literature focuses on the degree to which individuals are aware of the nudge’s presence and on the degree to which it influences deliberate and conscious decision-making processes [25,49,50]. Most often, nudge effectiveness regarding food choice remains the same regardless of whether an individual is aware of the nudge [24,36,51]. In addition, disclosing the rationale behind the nudge (and consequently making individuals aware of its presence) did not change the nudge’s effectiveness [52]. For example, a field experiment assessing a nudge’s effectiveness in increasing healthy snack choices (by repositioning healthy snacks) found the nudge to be effective regardless of whether the individuals were aware of its purpose [51]. Awareness of the nudge did not add, enhance, or decrease its effectiveness. Additionally, the acceptance of this nudge remained high across the different conditions [51]. Research further suggests that the effects of being aware of a nudge can vary from type to type, and more research is needed [36]. Thus, while informing individuals about the presence of a repositioning nudge does not change the nudge’s effectiveness, the results may be different for a priming nudge, such as the Giacometti cue. It has been suggested that being aware of the Giacometti cue may lead to reactant behavior [30,34]. Reactance may then lead to behavior opposite of what was intended—in our case, making high-calorie food choices. Reactance has been suggested as a tentative reason why the Giacometti cue was ineffective in a controlled field setting [53]. While making individuals aware of a nudge usually does not detract from its effectiveness, the opposite has been suggested for the Giacometti cue. Accordingly, we formulate research question five: (5) What is the role of the level of awareness of the Giacometti cue in actual food purchases in a real-world university cafeteria? It is important to test this research question in a real-world field setting assessing the level of awareness after the nudge has had its impact on preventing any confounding effects. Even though disclosing a nudge did not reduce its high acceptance level [51], different findings can be expected for the Giacometti cue because it did not achieve high acceptance ratings in an earlier study [48]. Consequently, we formulate our sixth research question, which to the best of our knowledge, has not been assessed so far: (6) What is the combined immediate effect of Giacometti cue acceptance and awareness on actual food purchases in a real-world university cafeteria?

**Figure 1:**
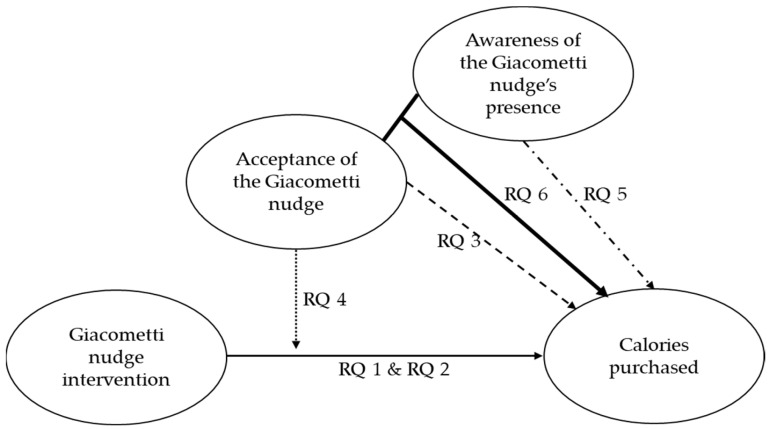
Overview of the present research model showing the research questions (RQ).

In our present study, we take up the call for more research assessing the effects of nudges on food choice in real-world field settings [19]. We apply a one-group pretest–posttest design in which the Giacometti cue serves as a nudge intervention to assess the following two aims: (1) Understand the effects of this cue on the actual food purchase of students in a complex real-world setting (a target group that has not yet been exposed to this cue), and (2) shed light on the working mechanisms of this cue regarding nudge acceptance and awareness. With these aims, we contribute to the understanding of when the Giacometti cue is effective for university students (research questions 1: during exposure and research question 2: after removal of the cue). The first two research questions test the validity of the nudge’s definition and that it needs to be present within the context to be effective [18]. In assessing the roles played by two influential factors (research questions 3 and 4: nudge acceptance; research question 5: nudge awareness; and research question 6: interaction between acceptance and awareness), we contribute to explaining under which conditions the Giacometti cue works in a real-world setting. In this, we test the Nudge Acceptance Model [42] and answer the call for more research regarding awareness of the Giacometti cue’s presence [15]. Thus, we can draw clear inferences regarding the theoretical background of nudging while testing its effectiveness in the real-world. An overview of our research model is presented in Figure 1.

## 2. Materials and Methods

### 2.1. Setting

Data were collected at a university cafeteria in North Rhine-Westphalia (Germany) from 10 October to 28 October 2022 at lunch time. We collected data Monday through Friday from approximately 11:45 a.m. to 1:45 p.m. This period marks the beginning of the new semester for the students. The cafeteria has two floors offering different dishes with adjacent seating areas. The ground floor cafeteria offers freshly made pizza and pasta as well as specialty dishes (for example, a vegan risotto). The first-floor cafeteria offers more common meat or fish dishes, vegetarian dishes, vegan dishes, and soup (for example, lasagna or pea soup). A variety of side dishes (for example, potatoes or carrots) is only offered on the first floor. Both cafeterias offer desserts and include a large salad bar. Table A1 in Appendix A exemplifies the various dish choices of an ordinary day. The main dishes offered on both floors can be considered equal regarding price and caloric value. On both floors, meat or fish, vegetarian, or vegan main dishes are offered for approximately the same prices. We only collected data on the first floor of the cafeteria because the ground floor cafeteria was closed during the pretest week due to a shortage of staff. This issue is discussed in the limitations section. Usually, about 2500 daily customers (mainly students) purchase and consume their lunch at this cafeteria. The three data collection weeks can be described as comparable. No exams took place during any of the weeks, the weather was comparable, and no special circumstances within the near vicinity of the cafeteria occurred.

### 2.2. Design and Procedure

All subjects gave their informed consent to inclusion before they participated in the study. This study was conducted in accordance with the Declaration of Helsinki, and the protocol was approved by the Ethical Review Committee Inner City Faculties (ERCIC) of Maastricht University (ERCIC_368_26_06_2022). For this real-world field setting, we used a one-group pretest–posttest design over a period of three consecutive weeks. The first week served as the pretest measure. In the second week, the nudge was introduced as an intervention, and in the third week (posttest), the nudge was removed. The nudge was placed in the entrance hall of the cafeteria under the two displays showing the dish options of the day. It was also placed on every counter where the participants chose their dishes (see Section 2.4 Materials). Research in real-world field settings qualifies as quasi-experimental research in which randomization is not possible as it would be in a controlled laboratory [39]. In assessing actual behavior that is unconfounded by the artificiality of a controlled laboratory setting, findings-based quasi-experimentation is considered valid and interpretable. Comparing behavior changes between different data collection points (observation 1: before the intervention, observation 2: during the intervention, and observation 3: after removal of the intervention) can be interpreted as induced by an intervention [39]. Thus, the present research design yields interpretable findings in a real-world setting. Such a one-group pretest–posttest design is often used to evaluate the effectiveness of interventions [54] and has been used previously in a field study on a visibility nudge in a university cafeteria [37]. This author compared the baseline and intervention weeks to determine the immediate effects of the nudge as well as the baseline and posttest weeks to determine the effects of the nudge after it had been removed from the setting. Field studies involve strengths and weaknesses that will be discussed in the limitations section.

Data were collected by a team of five female researchers between the ages of 20 and 35. They all dressed unobtrusively and were of average height and weight. Visitors to the cafeteria usually enter the cafeteria on the ground floor, automatically approaching the menu displays that show the daily meal choices for both floors of the cafeteria. Then, they decide whether to purchase their meal on the ground floor or climb the stairs to the first floor. Next, they approach one of the different food counters, choose their specific dish, purchase it, and sit down in the adjacent seating area to consume their meal. During data collection, customers were approached at random by one of the researchers shortly after they took their seats and started eating their meals. The researchers asked the individuals to participate in a short study about their meal choices made in the cafeteria that day, explained the procedure, and asked the customers to confirm their participation after giving their informed consent. The researcher then noted the participant’s meal choice (main dish(es), side dish(es), and dessert(s)) and asked the participant to complete the remaining questionnaire themselves. After filling out the questionnaire, participants inserted the questionnaire into an envelope provided by the researcher. Participants could only participate once. The research team ensured this prior to participation by specifically asking whether individuals had participated before. However, in the posttest week, the participants were allowed to participate again to achieve a group size comparable to that of the pretest and intervention weeks.

### 2.3. Participants

Participants were students and staff (usually postgraduate students) of the university having their lunch at the cafeteria. A priori power analysis (G*Power) revealed a required total sample size of *N* = 1548 to achieve a statistical power of 0.95 to detect an effect size of Cohen’s *f* = 0.10 [55]. This expected effect size is based on the effect size found for the effects of the Giacometti cue and nudging in general [32,56]. In total, *N* = 2899 participated in the study. Of these, 1407 (48.9%) were male, 1451 (50.4%) were female, and 21 (0.7%) were gender-diverse. In total, 2601 (94.8%) students participated, while 128 (4.7%) reported that they were faculty members. Only 16 (0.6%) participants were external to the university. On average, participants were *M* = 22.08 (*SD* = 3.77) years old. They were *M* = 175.9 cm (*SD* = 9.70) tall and weighed *M =* 68.5 kg (*SD* = 11.99) on average. The specific values per data collection week are displayed in Table 1 (see Section 3 Results). Participants in the three data collection weeks differed in gender, age, and weight (see Section 3 Results). More participants in the intervention week reported being gender-diverse than in the pretest and posttest weeks and were slightly older. Participants in the intervention week also weighed slightly more than participants in the posttest week. Participants indicated their motives for choosing their meal by checking all motives that applied. These motives can be ranked as follows: 1, need and hunger (*N* = 2010, 69.33%); 2, liking (*N* = 1830, 63.13%); 3, price (*N* = 1415, 48.81%); 4, convenience (*N* = 928, 32.01%); 5, health (*N* = 814, 28.08%); 6, sociability (*N* = 696, 24.01%); 7, habits (*N* = 614, 21.12%); 8, pleasure (*N* = 397, 13.69%); 9, visual appeal (*N* = 318, 10.97%); 10, natural concern (*N* = 137, 0.05%); 11, traditional eating (*N* = 110, 0.04%); 12, weight control (*N* = 90, 0.03%); 13, affect (*N* = 73, 0.03%); 14, social image (*N* = 45, 0.02%); and 15, social norm (*N* = 34, 0.01%). Comparisons of individuals who participated in the posttest week regarding their number of participations revealed that individuals who participated once were slightly older (*M* = 22.4; *SD* = 3.40) than individuals who participated twice (*M* = 21.26; *SD* = 2.70), with *t* (960.08) = 5.84, *p* < 0.001. Second participation in the posttest week likewise affected neither the acceptance of the Giacometti cue (*t* (961) = −0.481, *p* = 0.637) nor the number of calories purchased (*t* (854) = −0.687, *p* = 0.492) (Table A2 in Appendix B).

### 2.4. Materials

**Figure A1:**
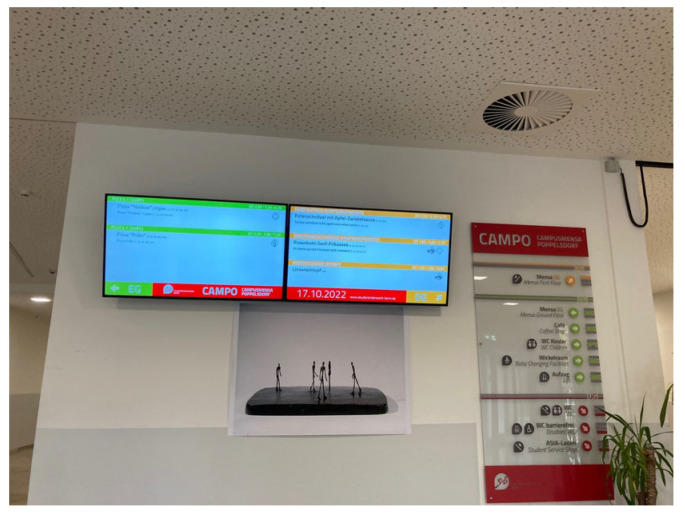
Exemplary placement of the Giacometti cue, Piazza, during the intervention week. The original work can be found at https://www.guggenheim.org/artwork/1426 (accessed on 27 April 2023).

**Figure A2:**
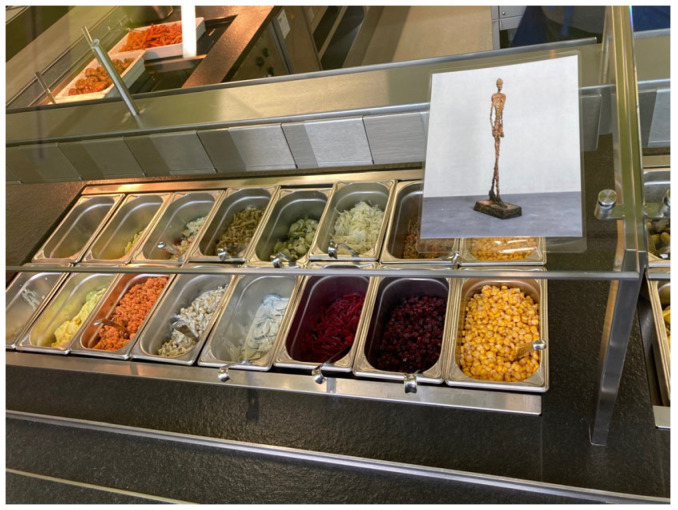
Exemplary placement of the Giacometti cue, L’homme qui marche, during the intervention week. The original work can be found at https://www.giacometti-stiftung.ch/highlights/objekt/?tx_artcollection_single%5Bartpiece%5D=419&cHash=beff4c349886757dc16117ec4da72324 (accessed on 27 April 2023).

In the intervention week, the participants were exposed to a nudge intervention in the form of posters displaying sculptures by the artist Alberto Giacometti [31]. The intervention was placed at all prominent places in the cafeteria where individuals usually make food choices (Supplementary Material File S1: layout of the cafeteria). In the entrance hall, directly under the displays showing the dish choices of the day, we placed a diagonal DIN A0 poster of the sculptures called Piazza (Figure A1 in Appendix C). At each counter on both cafeteria floors, we placed a DIN A5 poster of the sculpture called L’homme qui marche (Figure A2 in Appendix C). Both Giacometti cues had been previously used in other studies [15,30,32,33,34,57]. They have been found to be effective when applied in different formats, such as a screensaver [30], a DIN A0 poster [33], and a small sticker [34]. Its implementation in a cafeteria is not likely to disrupt the workflow and work processes of the cafeteria staff because it needs only be implemented once. For the practical implementation of nudges, it is important that a nudge does not inhibit the workflow within the setting where it is applied [19].

### 2.5. Measures

In the present study, we used a structured interview with a questionnaire. First, the researcher assessed the meal choice of the participant by means of an interview question. The researcher asked the participant what their meal choice was and noted the answer on a questionnaire. The researcher made sure that the entire meal choice was assessed and specifically asked the participant to indicate their choice of main dish(es), side dish(es), and dessert(s). Any additions to the meals (like ketchup, salad dressing, piece of bread, etc.) were also noted. If participants chose a salad from the salad bar as a main dish, the researchers categorized it according to its ingredients as vegan (green salad with vegetables), vegetarian (green salad with cheeses, etc.), and carb (green salad with couscous, potatoes, etc.). A combination of these categories was also possible. In addition, the researcher noted the data collection week (1 = pretest, 2 = intervention, and 3 = posttest) and the date and time of the questionnaire. Second, the researcher passed the questionnaire to the participant with a request for them to complete the questionnaire themselves to ensure anonymity regarding biometric data.

The questionnaire consisted of several sections. First, the participants’ motives for choosing their meals were assessed by checking all relevant motives. A list of 15 motives was provided based on The Eating Motivation Survey [58]. Second, participants indicated their level of hunger while choosing their meal on a 5-point scale ranging from not hungry at all to very hungry. Next, they rated their level of acceptance of nine different types of nudges on a 5-point scale ranging from do not agree to agree. This scale had been previously used in research on nudge acceptance [48,59]. We found a Cronbach’s alpha value of 0.694. The nine nudges are the messenger nudge, incentive 1 nudge, incentive 2 nudge, norms nudge, default nudge, salience nudge, priming nudge, affect nudge, and the Giacometti cue. Table A3 (Appendix D) shows the exact wording used to assess acceptance in German and English. While the acceptance of the Giacometti cue was of primary interest (I think it would be acceptable to advertise vegetable consumption in the cafeteria using posters on which skinny artistic sculptures are displayed), we added the acceptance of the other nudges mainly to report this information to the person in charge of food provision at the cafeteria. In assessing the acceptance of all nine different types of nudges, we used the same scale and the same items throughout all three weeks. Thus, the acceptance of the Giacometti cue was assessed in all three weeks in the same way. We intentionally described the nudge as a skinny artistic sculpture, not mentioning the name of the sculpture or the artist. In this way, we ensured that students who knew either the artist or sculpture would not answer differently than students who did not know the artist or sculpture. Finally, the participants indicated whether they were sitting in a group or alone and answered several demographic questions (age, height in cm, weight in kg, and gender). They were also asked to indicate their affiliation with the university (student, faculty member, or external) and state the number of times they had purchased a meal in this cafeteria in the current week (1–5 times). Only in the intervention week, when the nudge was present, were participants asked to assess their level of awareness regarding the Giacometti posters on a 5-point scale (not at all to very) after indicating their acceptance of the Giacometti cue (Supplementary Material File S2: questionnaire intervention week). We specifically asked how strongly the participants consciously perceived the posters in the cafeteria that day. To prevent any confounding effects of asking about the participants’ awareness of the presence of the cue, we did not portray a picture of the cue on the questionnaire, and we assessed nudge acceptance before awareness.

The dependent variable in this study was the number of calories purchased in the main dish(es). For each main dish, a caloric value was calculated based on the recipe provided by the cafeteria staff and a table of nutritional values [2]. Since some participants purchased two main dishes, we added the caloric values for all main dishes per participant. Main dishes normally do not include side dishes. Side dishes must be chosen separately. Thus, the caloric value of the main dishes purchased does not include the number of calories from any side dishes. The cafeteria usually offers around seven different main dishes per day to choose from. This was the case in the intervention week (number of main dishes ranging from 6–8) and the posttest week (number of main dishes ranging from 4–9). In the pretest week, the ground floor counters remained closed, and only about four different main dishes per day were offered that week (the number of main dishes ranging from 4–5). The following describes the average number of calories contained in the various main dishes offered per week: In the pretest week, main dishes ranged in their caloric value from 214 calories (pan-fried white cabbage, carrots, and peppers) to 1063 calories (currywurst with French fries) yielding an average count of 296 calories. In the intervention week, the main dishes contained, on average, 591 calories, ranging from 213 calories (pea soup) to 1063 calories (currywurst with French fries). In the posttest week, main dishes ranged in their caloric value from 213 calories (pea soup) to 1063 calories (currywurst with French fries), yielding an average number of 554 calories.

### 2.6. Analysis

As described in the measures section, the caloric values of the main dishes purchased were calculated for each participant. The cafeteria staff provided detailed recipes for the different main dishes, including the ingredients, exact measurements as well as portion size. The caloric value for every single dish offered was calculated based on a table of nutritional values using this information [2]. This caloric value was then assigned to the participants.

To answer the first (what is the immediate effect of the Giacometti cue on actual food purchases in a real-world university cafeteria?) and second research question (how does the removal of the Giacometti cue from a real-world university cafeteria affect the actual food purchases?), we conducted a univariate ANOVA on the number of calories purchased with main dishes using the data collection weeks as the independent variable. Bonferroni post hoc tests were applied to assess differences in the number of calories regarding the data collection weeks. To test the immediate effects of the Giacometti cue (research question 1), we considered the Bonferroni post hoc test comparing the pretest week and the intervention week because, by definition, a nudge has to be present within the decision-making context to be effective [18]. This approach has been used in earlier studies on nudges [37,51]. To test the effect of the Giacometti cue after its removal (research question 2), we considered the Bonferroni post hoc test, comparing the pretest week and the posttest week. This is the established analysis in a one-group pretest–posttest design [54] and has also been used before in a study on persistent nudge effects [37].

Research questions 3, 5, and 6 involve the number of calories purchased as a dependent variable as well as the acceptance of the Giacometti cue and the awareness of the Giacometti cue as independent variables. Because of a positive skewness of nudge acceptance (1.96; *SE* = 0.079) and nudge awareness (2.10; *SE* = 0.079), we computed groups based on the participants’ levels of acceptance and awareness of the Giacometti cue. Regarding the acceptance, participants were split into two groups: acceptance values of 1 qualified as low acceptance, and values of 2–5 qualified as high acceptance (*n_low_* = 634; *n_high_* = 264). This is based on the distribution pattern of Giacometti cue acceptance. The same procedure was performed for awareness of the Giacometti cue (*n_low_* = 697; *n_high_* = 201): awareness values of 1 qualified as low awareness, and values of 2–5 qualified as high awareness. Next, we conducted a univariate ANOVA to test these research questions. Only the number of calories purchased during the intervention week was considered in this analysis because the nudge needs to be present within the decision-making context to be effective [24].

To answer the third research question (what is the role of acceptance of the Giacometti cue on actual food purchases in a real-world university cafeteria?), we considered the main effect of the Giacometti cue acceptance on calories purchased. To answer the fifth research question (what is the role of the level of awareness of the Giacometti cue in actual food purchases in a real-world university cafeteria?), we considered the main effect of the awareness of the Giacometti cue’s presence on calories purchased. To answer the sixth research question (what is the combined immediate effect of Giacometti cue acceptance and awareness on the actual food purchases in a real-world university?), we considered the interaction effect of acceptance and awareness on calories purchased.

To answer research question 4 (does acceptance of the Giacometti cue moderate the nudge’s immediate effect on actual food purchases in a real-world university?), we conducted a moderation analysis using the PROCESS v 4.0 macro for SPSS developed by Andrew F. Hayes [60]. In the analysis, the data collection week represented the predictor, the dummy coded variable of the Giacometti cue’s acceptance was the moderator variable, and the number of calories purchased in main dishes was the outcome variable. Again, we only considered the pretest and intervention week in this analysis because a nudge needs to be present in the decision-making context to be effective [24]. We used a significance level of 0.05 and bootstrapping with 10,000 bootstrap samples for the percentile bootstrap confidence intervals (confidence level of 95%).

For all the analyses, we used SPSS v. 28 (IBM SPSS Statistics for Windows, version 28.0. Armonk, NY, USA, IBM Corp.), applied a significance level of 0.05, and deleted missing values listwise.

## 3. Results

The descriptive and inferential statistics for the three points of data collection are summarized in Table 1.

**Table 1:** Descriptive statistics for each data collection week and inferential statistics comparing the weeks.

		Pretest (*n* = 957)	Nudge Intervention (*n* = 968)	Posttest (*n* = 974)	Inferential Statistics
Gender	Male	470 (49.6%)	487 (50.7%)	450 (46.4%)	χ^2^ (2) = 11.79, *p* = 0.019
	Female	476 (50.2%)	468 (48.7%)	507 (52.3%)	
	Gender-diverse	2 (0.2%)	6 (0.6%)	13 (1.3%)	
Age		21.86 (3.59)	22.47 (4.42)	21.91 (3.16)	*F* (2, 2875) = 7.77, *p* < 0.001
Height		176.1 (9.71)	176.1 (9.68)	175.4 (9.71)	*F* (2, 2840) = 1.55, *p* = 0.213
Weight		68.6 (11.59)	69.4 (12.59)	67.6 (11.71)	*F* (2, 2727) = 5.05, *p* = 0.006
Hunger		4.16 (0.80)	4.15 (0.79)	4.12 (0.82)	*F* (2, 2888) = 0.858, *p* = 0.424
Number of Calories Purchased in Main Dishes		386.0 (193.59)	363.9 (168.42)	432.7 (178.19)	*F* (2, 2666) = 33.33, *p* < 0.001
Acceptance of the Giacometti Cue		1.46 (0.97)	1.58 (1.077)	1.43 (0.90)	*F* (2, 2880) = 5.766, *p* = 0.003
Nudge Awareness ^1^		-	1.54 (1.166)	-	-

Note: *SD* in brackets for all variables except gender. ^1^ nudge awareness was only assessed in the intervention week. Control variables are displayed above the dotted line.

There are no significant differences between the data collection weeks regarding height, overall nudge acceptance, and hunger. Regarding gender, the frequencies of gender-diverse individuals differ between the data collection weeks. There are slightly more gender-diverse individuals in the posttest week than in the pretest and intervention week. We find significant differences between the data collection weeks for age. Bonferroni post hoc test revealed that participants in the intervention week are slightly older than participants in the pretest week (*p* < 0.001) and in the posttest week (*p* = 0.001). Participants in the pretest and posttest weeks do not differ in age (*p* = 0.786). There are significant differences in participants’ weights between the data collection weeks. Participants in the intervention week weigh slightly more than participants in the posttest week (*p* = 0.002). There are no differences in weight between participants in the pretest and intervention week (*p* = 0.157) or between the pretest and posttest weeks (*p* = 0.081). Regarding Giacometti cue acceptance, there is a significant difference between the data collection weeks. Participants in the intervention week accept the Giacometti cue more readily than participants in the pretest week (*p* = 0.012) and participants in the posttest week (*p* = 0.001). There is no difference in Giacometti cue acceptance between the pretest and posttest weeks (*p* = 0.489). Considering all data collection weeks, we find significant correlations of gender with acceptance of the Giacometti cue (*r* = −0.189, *p* < 0.001) as well as of acceptance and numbers of calories purchased (*r* = −0.157, *p* < 0.001). For a simpler interpretation of these results and due to the small number of gender-diverse individuals in this sample, we only considered males and females. These correlations indicate that female participants accept the Giacometti cue less readily and purchase fewer calories. We also find a positive correlation between acceptance of the Giacometti cue and level of awareness of this nudge (*r* = 0.120, *p* < 0.001). The more aware the participants were of the nudge, the more they accepted it.

A univariate ANOVA reveals a significant main effect of the data collection weeks on the number of calories purchased with main dishes (*F* (2, 2666) = 33.330, *p* < 0.001; *R*^2^ = 0.024; ƞ^2^ = 0.024). The Giacometti cue has a small effect and explains about 2% of the variance in the number of calories purchased. To answer research question 1 (what is the immediate effect of the Giacometti cue on actual food purchases in a real-world university?), we considered the Bonferroni post hoc test comparing the pretest and the intervention week. The average of calories purchased in the intervention week (*M* = 363.90; *SD* = 168.42) is significantly lower than the average of calories purchased in the pretest week (*M* = 385.96; *SD* = 193.59), *p* = 0.028. We conclude that the Giacometti cue significantly and immediately reduces the number of calories purchased. 

To answer research question 2 (how does the removal of the Giacometti cue from a real-world university cafeteria affect the actual food purchases?), we considered the Bonferroni post hoc test comparing the pretest and posttest weeks. The average of calories purchased in the pretest week (*M* = 385.96; *SD* = 193.59) is significantly lower than the average of calories purchased in the posttest week (*M* = 432.71; *SD* = 178.19), *p* < 0.001. To ensure that the second participation of participants in the posttest week did not confound the results for the second research question, we repeated the analysis filtering out individuals who participated for a second time. The results remain the same with *F* (2, 2290) = 20.509, *p* < 0.001, and a Bonferroni post hoc test with *p* < 0.001. The average of calories purchased in the pretest week (*M* = 385.96; *SD* = 193.59) is significantly lower than the average of calories purchased in the posttest week (*M* = 429.11; *SD* = 178.87), only considering individuals who participated once in the study. We conclude that participants purchased more calories after the nudge had been removed.

To answer research question 3 (what is the role of acceptance of the Giacometti cue in actual food purchases in a real-world university cafeteria?), we considered the main effect of nudge acceptance on the number of calories purchased in the intervention week. The univariate ANOVA reveals a significant main effect of the acceptance of the Giacometti cue (*F* (1, 894) = 4.717, *p* = 0.030; ƞ^2^ = 0.005). Individuals with a high acceptance of the Giacometti cue purchase more calories (*M* = 385.54; *SD* = 189.22) than individuals with a low Giacometti cue acceptance (*M* = 354.88; *SD* = 159.09). This effect is considered small. We conclude that those participants who readily accepted the Giacometti cue purchased more calories than participants who did not accept the cue.

To answer research question 4 (does acceptance of the Giacometti cue moderate the nudge’s immediate effect on actual food purchases in a real-world university?), the moderation analysis considering data from the pretest and intervention week finds no significant interaction between the data collection weeks and acceptance of the Giacometti cue (*b* = 32.16; *SE* = 19.30; *t* = 1.667, *p* = 0.096; 95% CI [−5.690 to 70.011]). The model is significant with *F* (3, 1798) = 4.008 and *p* = 0.007 (*R*^2^ = 0.007). Acceptance of the Giacometti cue does not moderate the effect of the nudge on the number of calories purchased. We conclude that accepting the Giacometti cue more or less does not impact the number of calories purchased.

To answer research question 5 (what is the role of the level of awareness of the Giacometti cue in actual food purchases in a real-world university cafeteria?), we considered the main effect of nudge awareness on the number of calories purchased in the intervention week. The univariate ANOVA does not show a significant main effect (*F* (1, 894) = 0.321, *p* = 0.571; ƞ^2^ = 0.000). We conclude that individuals with a high versus a low awareness of the Giacometti cue do not differ in their number of calories purchased.

To answer the sixth research question (what is the combined immediate effect of Giacometti cue acceptance and awareness on actual food purchases in a real-world university?*)*, we considered the interaction effect between nudge awareness and nudge acceptance on the number of calories purchased. The univariate ANOVA does not show a significant interaction effect (*F* (1, 894) = 0.048, *p* = 0.827; ƞ^2^ = 0.000). There is no combined effect of these variables on the number of calories purchased while the nudge was present. Students high or low in nudge acceptance do not differ from students high or low in nudge awareness regarding the number of calories purchased. The corresponding descriptive statistics (Table 2) show that most students (57.6%) have low acceptance and also awareness ratings. This group of students purchased the smallest number of calories. In contrast, only a few students (9.4%) show high acceptance as well as awareness ratings. These students purchased the largest number of calories. We conclude, descriptively speaking, that students who did not accept the nudge and were unaware of its presence purchased the least calories, while students who accepted the nudge and were aware of its presence purchased the most calories.

The model testing research questions 3, 5, and 6 explain about 1% of the variance in the number of calories purchased (*R*^2^ = 0.007).

## 4. Discussion

The present study contributes to bridging the gap in research assessing the effects of nudges on food choice in real-world settings [19]. In testing the real-world effectiveness of the Giacometti cue regarding the new target group of university students, we add to the ecological validity of prior findings and, thus, the generalizability of this cue. By researching what happens when the Giacometti cue is removed from the setting, we gain insights regarding the nudge’s validity. In assessing the roles played by acceptance and awareness, we deepen our understanding of the conditions under which nudges can be effective. We found the Giacometti cue to be immediately effective in reducing the number of calories purchased (research question 1). When it was removed from the decision-making context, it had a reversal effect, increasing the number of calories purchased (research question 2). In addition, individuals with a high acceptance of the Giacometti cue purchased more calories than individuals with a low acceptance (research question 3). The effect of the Giacometti cue was not influenced by the extent to which the participants accepted the Giacometti cue (research question 4). Being more or less aware of the Giacometti cue’s presence did not affect the number of calories purchased (research question 5). Comparing students with high versus low nudge acceptance with students with high versus low awareness yielded no significant findings regarding the number of calories purchased.

### 4.1. Effects of the Giacometti Cue

Our first two research questions focus on the effects of the Giacometti cue during exposure and after its removal in a real-world field setting. Research on the Giacometti cue found it to effectively improve the dietary behavior of adults ranging between 35 and 39 years of age with a moderate effect size [30,32]. For young adults, the Giacometti cue was ineffective when applied in a virtual setting [53,57]. In the present study, the Giacometti cue had an immediate effect of reducing the number of calories purchased by university students in a real-world university cafeteria. This is in line with the results of most of the studies assessing the Giacometti effect, e.g., [30], and also with other studies regarding immediate nudge effects in cafeteria settings, e.g., [37]. Based on the literature regarding the effectiveness of the Giacometti cue so far, its immediate effect of reducing the number of calories purchased was expected (research question 1).

In addition to immediate effects, some types of nudges established effects that lasted even after the nudge had been removed from the decision-making context [36,37]. For example, a visibility nudge in a university cafeteria still had the intended lasting effect of increasing the number of vegetarian dishes sold after they had been removed [37]. Such lasting effects of nudges are believed to depend on the cognitive processes that the nudge targets [38]. So far, only one study has assessed the longitudinal effects of a six-month exposure to the Giacometti cue in approximately 48-year-old individuals with weight loss goals [34]. They found that specifically restraint eaters aware of the nudge lost weight after the six-month-long exposure, showing that the Giacometti cue does not lose its efficacy during long-term exposure. This study did not assess any effects after the removal of the nudge [34]. Based on research findings so far, it can be assumed that the Giacometti nudge either shows a lasting effect of reduced numbers of calories purchased or non-significant results. However, in the present study, the number of calories purchased by students increased after the nudge had been removed from the decision-making context. It seemed to have a lasting effect; however, according to earlier research and the purpose of the nudge, this increase was not expected. The evidence on the Giacometti cue so far has not predicted an increase in the number of calories purchased [33,34]. The results of our study, therefore, support the call for research on the long-term effects of nudges [26,44]. Similar unintended effects of nudges have been reported regarding food choices [61,62]. One study found young adult interns in a workplace cafeteria to unexpectedly reduce their healthy food choices after having been nudged by personalized e-mails and green footsteps on the floor. It has been suggested that these nudges may have been regarded as overly intrusive and paternalistic to be acceptable [29]. Such defiance arousal has been described as one reason for the backfiring effects of health interventions [63]. A backfiring effect is an unintended negative intervention outcome causing the opposite effect of that intended by the intervention [63]. A possible explanation for the unexpected increase in calories purchased after the Giacometti cue had been removed is that it may have aroused defiance. Low acceptance ratings of this cue found in the present study, as well as previous research [48], indicate that it was not well received as a health intervention in general. Participants possibly counteracted by increasing the number of calories purchased after its direct exposure was removed. These findings point to the important role of nudge acceptance.

### 4.2. The Role of Nudge Acceptance

The present study researched the role of nudge acceptance by addressing two research questions: What is the role of acceptance of the Giacometti cue on actual food purchases in a real-world university cafeteria (research question 3)? Does acceptance of the Giacometti cue moderate the nudge’s effect on actual food purchases in a real-world university cafeteria (research question 4)? Considering the role of Giacometti cue acceptance, we found that individuals who readily accepted the nudge purchased more calories than individuals who did not accept the nudge when exposed to it (with a small effect). In addition, we found no moderation effect of nudge acceptance considering the difference in the number of calories purchased before nudge exposure and during nudge exposure. The level of nudge acceptance did not influence the relationship between the Giacometti cue and the number of calories purchased. These results were not expected—especially those involving higher numbers of calories purchased by individuals who accepted the nudge. To explain these results, we need to consider the Nudge Acceptance Model in more detail [42]. According to this model, the more a nudge is accepted, the more likely it is to be effective [42]. In our case, this means that those participants who readily accepted the Giacometti cue should have purchased fewer calories. Instead, they purchased more. Moreover, the immediate effect on the number of calories purchased (research question 1) should have been moderated by the level of nudge acceptance. We found no such effect. The Nudge Acceptance Model proposes that nudge acceptance, and subsequently behavior change, is influenced by the degree of transparency of the nudge [42]. Transparency encompasses whether or not a nudged individual correctly understands the purpose of the nudge as well as the intended behavior change [25,42]. Thus, for a nudge to be accepted and cause the intended behavioral change, the nudged individual needs to understand the purpose of the nudge. In the case of the present study, it is possible that the purpose behind the Giacometti cue was not transparent enough for the individuals to understand it correctly. The cue’s skinny body shape is intended to prime weight-related cues, which suggest weight loss, e.g., [30,34], leading to a reduction in calories purchased. Considering that the Giacometti cue depicts a particularly skinny (even underweight) body shape, it is possible that this cue did not activate thoughts of weight-loss in young university students but rather thoughts of weight-gain to counteract underweight. In this case, readily accepting the Giacometti cue and consequently purchasing more calories is logical. This explanation is strengthened when considering that an individual’s response to an intervention (including nudges) depends, for example, on an individual’s preferences [22,29]. These motives did not match the purpose of the Giacometti cue, looking at the individuals’ preferences regarding the reasons for choosing a particular meal in the present study. Students ranked weight control number twelve out of fifteen motives for choosing their meal (see Section 2.3).

So far, little is known about the relationship between nudge acceptance and nudge effectiveness in real-world settings [19]. Considering the Giacometti cue, we can conclude from the present study that transparency (thus correctly reconstructing the purpose behind a nudge) is necessary for this cue to have the intended effect. So far, the Giacometti cue has only been researched as displaying a skinny body shape without any explanatory information regarding its purpose. This Giacometti cue can benefit from modifications to improve its acceptance and ensure that its aims are correctly understood when applied to students in a real-world university cafeteria.

### 4.3. The role of Nudge Awareness

In research question 5, we proposed that the degree to which university students are aware of the Giacometti cue plays a role in their real-world food choices. Nudges have been described as influencing automatic and unconscious decisions as well as deliberate and conscious decisions [25]. Making someone aware of the nudge’s presence does not affect the effectiveness of nudges, e.g., [52], specifically so in a real-world setting [51]. As expected, and in line with this finding, the present study did not find the degree to which participants were aware of the Giacometti cue (high versus low awareness) to affect the number of calories they purchased when exposed to the nudge. In our study, we assessed nudge awareness after exposure to the Giacometti cue. We neither made them deliberately aware of the nudge nor did we disclose its purpose. We consider this approach as assessing the unconfounded level of awareness and not its perceived purpose. We focused on whether the students noticed the nudge in a hectic university cafeteria. Because the students were generally unaware of the Giacometti cue, and it effectively reduced the number of calories purchased when it was present within the setting, we can confirm that its influence was unconscious. This is in line with earlier studies on the Giacometti cue [15,30]. Thus, regarding the role of awareness, we can confirm earlier research suggesting that the Giacometti cue influences subconscious cognitive processes. It does not have to be consciously perceived to be effective and can therefore be applied in hectic real-world settings, such as university cafeterias.

In the sixth research question, we inquired about the combined effect of Giacometti cue acceptance and awareness of its presence in food purchases. This type of research question has not yet been explored in research on nudge effectiveness, and we found no combined effect in the present study. So far, disclosing the purpose of a nudge (thus making individuals aware of its presence) neither reduced nudge effectiveness on dietary behavior nor its acceptance when dealing with a highly accepted nudge [51]. As explained above, the Giacometti cue did not achieve good acceptance ratings in the present study nor in an earlier study involving university students [48]. In the present study, only 29.4% of students accepted this nudge. In addition, most students (77.6%) were not aware of it. We found differences in the number of calories purchased when the nudge was present, comparing individuals with high or low acceptance levels of the Giacometti cue. However, these differences did not vary in relation to the groups with high and low awareness of the Giacometti cue’s presence. These are positive results for the Giacometti cue. The level of acceptance did not change when students were aware of the Giacometti cue, and consequently, the number of calories purchased while the nudge was present did not change. Even though the combined effect of nudge acceptance and awareness was not significant, the descriptive statistics would suggest that the Giacometti cue may have its intended effect of inducing students to purchase fewer calories if they do not accept the nudge and are unaware of its presence. Students who accept this nudge and are aware of its presence may purchase larger numbers of calories. Again, this unexpected increase in calories purchased for individuals who were highly aware of the cue and readily accepted it can be explained by the lack of a clear statement regarding the nudge’s purpose (see Section 4.2). Still, we do not know if nudge awareness amounts to (mis)understanding its purpose. In this regard, we conclude that more research is needed to determine if the purpose of the Giacometti cue is correctly understood and whether the cue’s effectiveness benefits from making individuals aware of the cue and its purpose.

### 4.4. Methodological Reflections and Future Research

Study design: The present field study using a one-group pretest–posttest design was carefully designed based on well-established standards and previously used approaches [37,54]. A weakness of the field study design is that replicability is difficult [64]. These difficulties can be counteracted to ensure that valid data is obtained—for example, large sample size and measurements before and after the intervention [64]. Another weakness is that confounding variables may play a role in a field setting. This weakness can be counteracted by standardizing conditions and settings as much as possible [54]. A strong point of the present study is that we took the proposed measures to counteract any weaknesses: (1) To obtain valid data, we reached a large sample size and applied measurements before and after the intervention. Even though replicating the exact dish choices offered in the present study is difficult in another cafeteria setting, we ensured replicability by calculating the caloric value for each dish. This can be accomplished for any dish offered in any cafeteria setting. Future studies that aim at replicating our results should, therefore, also calculate the caloric values of the dishes offered in their setting. (2) We also took measures to keep the conditions as standardized and constant as possible [54]. Despite all this, during the pretest week, the ground floor cafeteria was closed due to an unforeseen staff shortage. Consequently, the range of meals, as well as the average calorie content of the main dishes, was smaller in the pretest week than in the other two weeks. While this is an unforeseen limitation of the study, it does not limit our findings, as we found that the students purchased fewer calories in the intervention week than in the pretest week (when the average number of calories per main dish was higher than in the pretest week). Future studies need to be aware of unforeseen events that can hinder the standardization of conditions.

Food choice and purchase: The dependent variable in the present study is the actual purchase of main dishes (converted into corresponding numbers of calories). This represents the number of calories students intended to consume as their lunch. A strong point of this measure is the relative ease of data collection which allowed for a large sample size to be assessed, increasing the generalizability of our findings. Because the subsequent calculations of the dishes’ caloric values were based on the exact recipes provided by the cafeteria staff, these values are very precise. Calories are often used as a dependent variable in research (also involving nudges) [22]. A weakness of this measure is that we do not know whether participants actually consumed everything they purchased. Because caloric values were based on dish recipes, these calculations are valid. However, low numbers of calories do not reflect a healthy diet, and we cannot draw clear conclusions in this regard. A lower caloric intake may be an important aspect of a healthy diet [2], but healthy eating also involves the consumption of, for example, high in nutrients and vitamins [2,65]. We want to clarify that we do not suggest that low-calorie food choices represent healthy eating behavior. Unobtrusively assessing actual food consumption in a real-world setting is difficult and hardly feasible for a large sample size. Still, future research should consider actual food consumption as a more precise measure, better reflecting the effects of the Giacometti cue in a real-world cafeteria.

Giacometti cues: In the present study, we presented sculptures designed by Alberto Giacometti that are known to display skinny, human-like figures. We applied these cues on posters in a busy university cafeteria. A strong point of using this cue is that it effectively improved snack choice when applied as a poster next to a vending machine in a university cafeteria [33]. Therefore, our chosen format is suitable. This artwork combines two lines of research in the field of food choice: the influence of body shape primes and the influence of external cues [30]. A weakness of this cue is that while it represents a skinny individual, it does not show real (or realistic) individuals. It can neither be described as aesthetic nor attractive from a conventional point of view. While the present study measured acceptance and awareness of the Giacometti cue, it did not assess in which way individuals perceived the cue. Its low acceptance rates may indicate a disliking of the cue. Research in the marketing domain found that art conveys positive connotations to unrelated products regardless of the specific content of the artwork [66]. Whether this is true regarding the Giacometti cue is yet to be determined. Future research should therefore focus on how the Giacometti nudge is perceived. Do individuals recognize it as art? Do they recognize the artist? Do they associate artistic pleasure with this sculpture, displeasure, or rather a weight prime?

Control variables: We assessed fifteen different motives for choosing one’s meal based on a validated questionnaire. A strong point of this procedure is that we learn more about the sample of university students and for what reasons they chose their meals within the real-world cafeteria setting. A weakness is that these reasons did not include veganism, vegetarianism, or food allergies. These reasons may have played a role in food choice. Future research should consider these reasons as control variables.

Constraints of time and resources: The present study was conducted over three consecutive weeks. A strong point of this approach is its feasibility and that this allows for a more standardized setting because it involves the collection of data over time. A weakness is that due to constraints of time and resources, we were not able to assess any long-term effects of the Giacometti cue by, for example, repeating the study after a certain period. Future studies should consider the assessment of possible long-term effects in their study design. The present study focuses on the Giacometti cue and its effects on food choice. A weakness of this focus is that we were not able to compare the found effects to other nudging cues. A strong point of this focus is again its feasibility. Adding a second cue or type of nudge would have expanded the data collection period. Still, comparing the effects of the Giacometti nudge to the effects of other cues and nudges is an interesting topic for future research.

Giacometti cue acceptance: To measure acceptance of the Giacometti cue, we asked the participants to indicate whether they accepted the portrayal of artwork showing skinny artistic sculptures. The strong point is that this measure was used in a previous study [48]. A weakness is that this question probes two aspects simultaneously—namely, skinniness and artwork. Because we did not want to confound the effect of the Giacometti cue, we did not show a picture of the nudge as an example. In addition, we intentionally did not include the name of the artist or sculpture in this assessment. An interesting subject for future studies is how the acceptance of the Giacometti cue is perceived when the artist and a picture are included. The acceptance of the Giacometti cue as a health intervention and its impact on nudge effectiveness remains a prominent topic for future research because the present findings in this regard were unexpected. The reasons for these findings should be empirically assessed. Moreover, other modifications to this cue may be beneficial. For example, future studies need to assess whether clearly stating the purpose behind this nudge (and therefore increasing its transparency) leads to the intended effect of higher nudge acceptance causing fewer calories purchased. So far, research found that transparency does not hinter a nudge’s effectiveness [28]. A study on the transparency of the Giacometti cue may explain our unexpected results regarding nudge acceptance. Future studies should also assess specifically how the target group of young adults perceived the purpose of the nudge. Did the cue initiate weight-loss or rather weight-gain associations in young adults?

### 4.5. Implications for Theory and Practice

Several implications regarding the theoretical background of nudging, as well as the practical application of nudges, can be drawn from this study. The Giacometti cue was applied in a real-world setting with the goal of improving the food choice of university cafeteria customers. While this implementation was easy, cost-effective, and led to fewer calories being purchased in the intervention week, its effect of increasing the number of calories purchased after it had been removed from the setting was unexpected. As a practical implication, these findings stress the importance of testing a nudge’s effectiveness even if it has been identified as suitable, easy to apply, and cost-effective. As a theoretical implication, the results show that a nudge may also have an effect even if it is removed from the setting—contrary to its definition [18].

Most individuals perceived the Giacometti cue as unacceptable. In addition, those individuals who consciously accepted it purchased the most calories. As a practical implication, the acceptance of any nudge should always be ascertained prior to its implementation. As a theoretical implication, these results show that regarding the Giacometti cue, another important factor of the Nudge Acceptance Model [42] is the understanding of the purpose behind the nudge (transparency). Its purpose needs to be correctly understood to have the intended effect. Future research is necessary to understand these specific findings. What do young adults associate with this cue—weight gain or weight loss?

Combining the just mentioned implications indicates that the Giacometti cue’s effectiveness can benefit from the refinement of its use in a real-world cafeteria targeting university students. These refinements should focus on ensuring that its purpose is correctly understood while increasing its acceptance. For example, adding a message that explains the purpose of the cue may increase its acceptance and transparency. Another way to increase its acceptance is to present actual skinny sculptures based on Giacometti’s artwork instead of posters. The nudge is then more likely to be perceived as genuine artwork. Our understanding of the Giacometti cue’s effects can also benefit from comparing its effects with those of another artwork nudge, which can be clearly associated with weight loss.

The level of awareness of the Giacometti cue’s presence did not change its acceptance nor did it change the number of calories purchased while present. As a practical implication, these findings indicate that the Giacometti cue can be applied in a real-world cafeteria setting without the need to focus customers’ attention on it. This is good news for highly frequented university cafeterias. As a theoretical implication, these findings underline research findings so far regarding the role played by awareness and transparency of a nudge [28].

## 5. Conclusions

The present study investigated the effects of the Giacometti cue on food choice in a real-world university cafeteria setting. The role played by nudge acceptance, and nudge awareness, as well as the combined effects of these influences, were also assessed. Based on our findings, we conclude the following: (1) The Giacometti cue has an immediate and intended effect of reducing the number of calories purchased when it is present in a real-world university cafeteria. (2) Unexpectedly, the removal of this cue led to an increase in calories purchased. This increase can possibly be explained by defiance arousal. (3) As individuals who readily accepted the nudge unexpectedly purchased more calories than individuals who did not accept the nudge, nudge acceptance plays an important role. This role is not fully understood and may be explained by a lack of transparency of the nudge’s purpose. (4) As expected, the Giacometti cue does not have to be consciously perceived to be effective in a real-world university cafeteria. Therefore, it is suitable for application in such a hectic environment.

The present findings have implications for the practical application of this cue in a real-world cafeteria as well as theoretical implications regarding the nudge definition and the Nudge Acceptance Model [42]—specifically regarding the importance of nudge transparency. Moreover, the Giacometti cue may benefit from modifications, which need to be assessed in future studies. More research is needed to understand the unexpected findings in the present study—specifically involving the question of whether making individuals aware of the nudge’s presence and purpose increases its acceptance and, consequently, its intended effectiveness in reducing the number of calories purchased.

## Figures and Tables

**Table 2:** Descriptive statistics of the interaction between Giacometti cue acceptance and awareness in the intervention week regarding the number of calories purchased.

		Acceptance of the Giacometti Cue			
		Low (*n* = 634)		High (*n* = 264)	
		*M* (*SD*)	*n* (%)	*M* (*SD*)	*n* (*%*)
Awareness of the Giacometti cue	Low (*n* = 697)	354.44 (156.54)	517 (57.6)	382.02 (196.53)	180 (20.0)
	High (*n* = 201)	359.36 (172.29)	117 (13.0)	393.10 (173.42)	84 (9.4)

## Data Availability

The data presented in this study are available upon request from the corresponding author. The data are not publicly available due to privacy reasons as biometric data was collected.

## Supplementary Materials

The following supporting information can be downloaded at: https://www.mdpi.com/article/10.3390/healthcare11091307/s1, Supplementary Material File S1: layout of the cafeteria; Supplementary Material File S2: questionnaire intervention week.

## Appendix A

**Table A1:** Sample menu from 17 October 2023 (Monday in the intervention week).

Dish Category	Dishes	Calories (Total)
Vegetarian/vegan	Brussel sprouts and mustard fricassee (vegan)	280
Meat/fish	Lemongrass tofu Vietnamese style with basmati rice (vegan)	624
	Turkey Schnitzel with apple-onion sauce	439
	Pasta with meat sauce	454
Vegetarian stew	Lentil soup (vegan, large portion)	224
	Lentil soup (vegan, small portion)	112
Pizza	Pizza margarita	579
	Pizza verdure (vegan)	549
Salad bar	Salad bar vegan (green salad with vegetables)	60
	Salad bar vegetarian (green salad with cheeses, etc.)	270
	Salad bar carb (green salad with couscous, potatoes, etc.)	210
	Salad bar vegan, carb (green salad with vegetables, couscous, potatoes, etc.)	195
	Salad bar vegan, vegetarian (green salad with vegetables, cheeses, etc.)	300
	Salad bar vegetarian, carb (green salad with cheeses, couscous, potatoes, etc.)	405
	Salad bar vegan, vegetarian, carb (green salad with vegetables, cheeses, couscous, potatoes, etc.)	435

## Appendix B

**Table A2:** Descriptive and inferential statistics regarding the individuals from the posttest week and their number of participations in this study.

		Participated Once (*n* = 547)	Participated Twice (*n* = 423)	Inferential Statistics
Gender	Male	248 (45.5%)	(47.5%)	χ^2^ (2) = 1.15, *p* = 0.563
	Female	288 (52.8%)	(51.5%)	
	Gender-diverse	9 (1.7%)	4 (1.0%)	
Age		22.41 (3.40)	21.26 (2.70)	*t* (960.08) = 5.84, *p* < 0.001
Height		174.9 (9.59)	176.04 (9.85)	*t* (954) = −1.758, *p* = 0.079
Weight		68.0 (12.01)	67.1 (11.34)	*t* (915) = 1.17, *p* = 0.243
Hunger		4.12 (0.82)	4.11 (0.83)	*t* (965) = 0.154, *p* = 0.878
Number of calories purchased in main dishes		428.88 (179.36)	437.33 (177.44)	*t* (854) = −0.687, *p* = 0.492
Acceptance of the Giacometti cue		1.42 (0.84)	1.45 (0.99)	*t* (961) = −0.481, *p* = 0.637

*Note*: *SD* in brackets for all variables except gender. Control variables are displayed above the dotted line.

## Appendix D

**Table A3:** German and English items that measure nudge acceptance.

Type of Nudge	Item
Messenger nudge German	Berühmte Personen als Informationsquelle
Messenger nudge English ^1^	Celebrities as a source of information
Incentive 1 nudge German	Wettbewerb zum größten Gemüseverzehr
Incentive 1 nudge English ^1^	Competition on the largest vegetable intake
Incentive 2 nudge German	Kampagnen mit abschreckenden Botschaften
Incentive 2 nudge English ^1^	Scare campaigns
Norms nudge German	Informationen zum Gemüsekonsum von Kommilitonen
Norms nudge English ^1^	Information on vegetable consumption of fellow students
Default nudge German	Grüner Salat als automatische Beilage (die auch abgewählt werden kann)
Default nudge English ^1^	Green salad as a default choice (which can easily be deselected)
Salience nudge German	Poster mit Tipps für einen höheren Gemüsekonsum
Salience nudge English ^1^	Posters with tips on how I could eat more vegetables
Priming nudge German	Ansprache durch Mensa-Mitarbeiter, die nach zusätzlicher Gemüse-Auswahl fragen
Priming nudge English ^1^	Staff asking about additional vegetable choices
Affect nudge German	Ansprechendere Bezeichnung von Gerichten mit viel Gemüse
Affect nudge English ^1^	More appealing names for dishes containing many vegetables
Giacometti cue German ^2^	Poster, auf denen sehr dünne künstlerische Skulpturen zu sehen sind
Giacometti cue English ^2^	Posters on which skinny artistic sculptures are displayed

*Note:* ^1^ The items in English [67] were translated into German. ^2^ These are based on prior work of the authors [48].
