# Supplementary material for: Effects of a Nudging Cue Targeting Food Choice in a University Cafeteria: A Field Study
Source: Healthcare (Basel). 2023 May 3;11(9):1307. doi: 10.3390/healthcare11091307 (PMC10178432; doi:10.3390/healthcare11091307)

Layout of the cafeteria and placement of the nudges: Ground floor

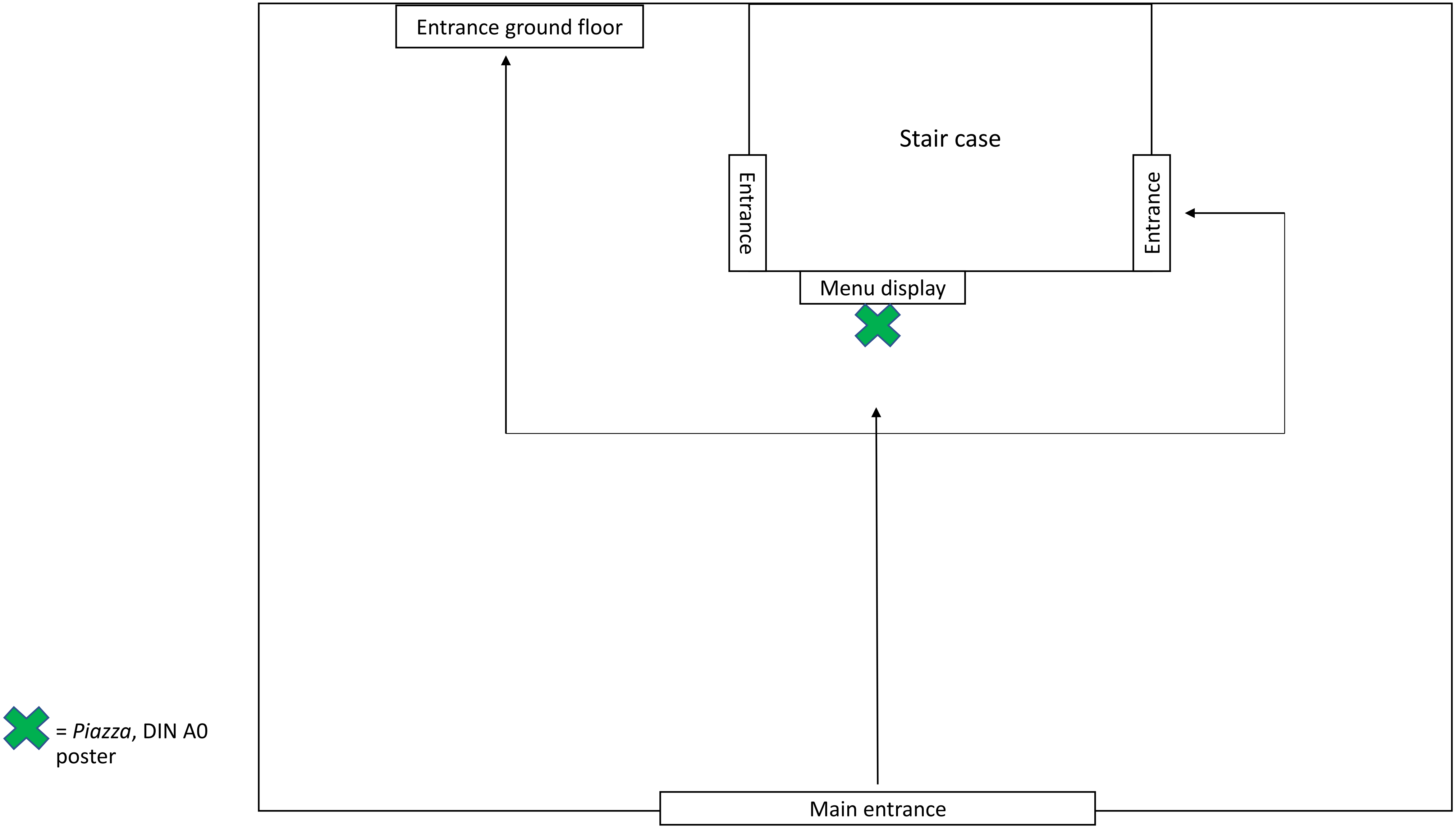

Layout of the cafeteria and placement of the nudges: Ground floor

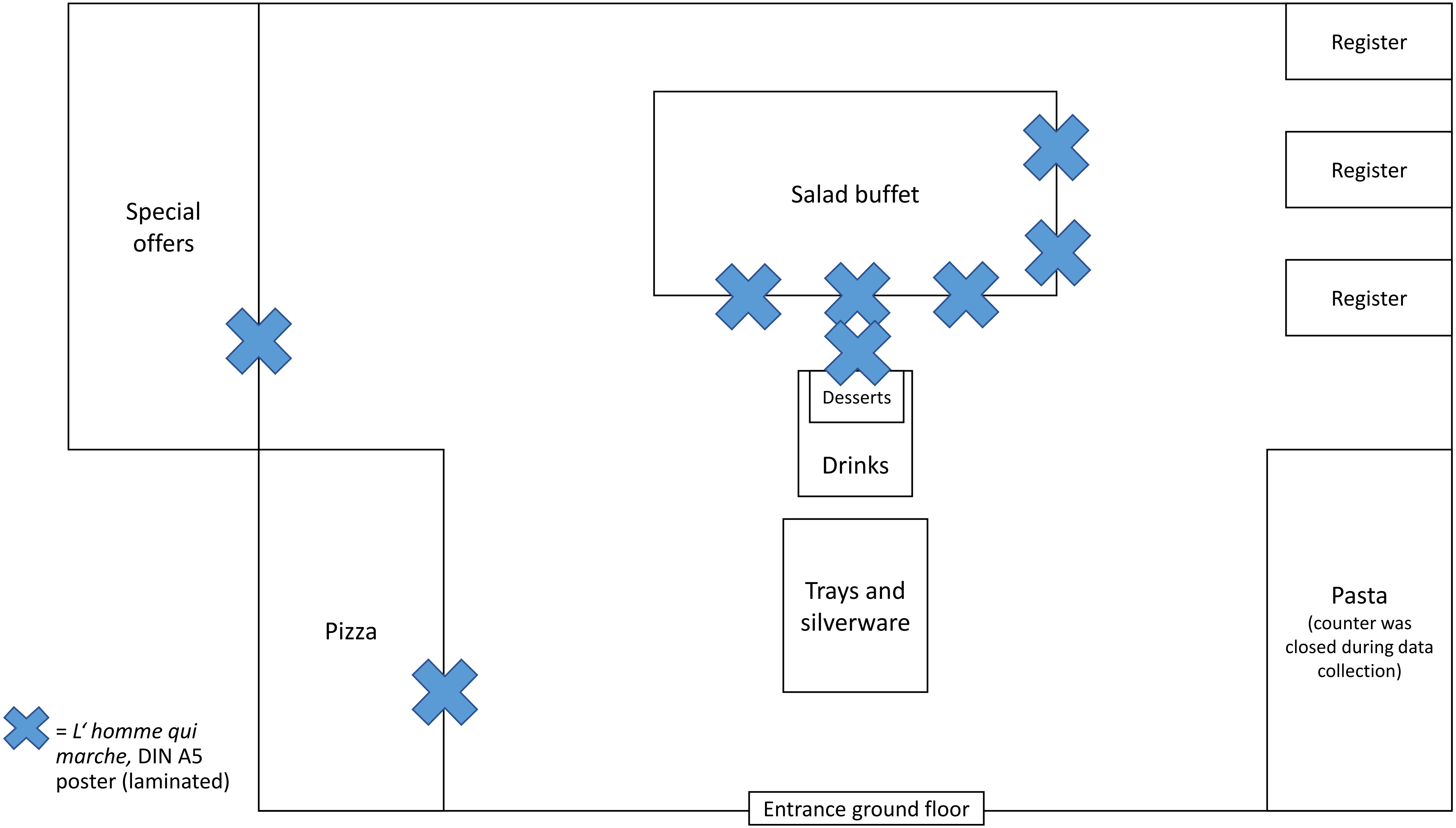

Layout of the cafeteria and placement of the nudges: First floor

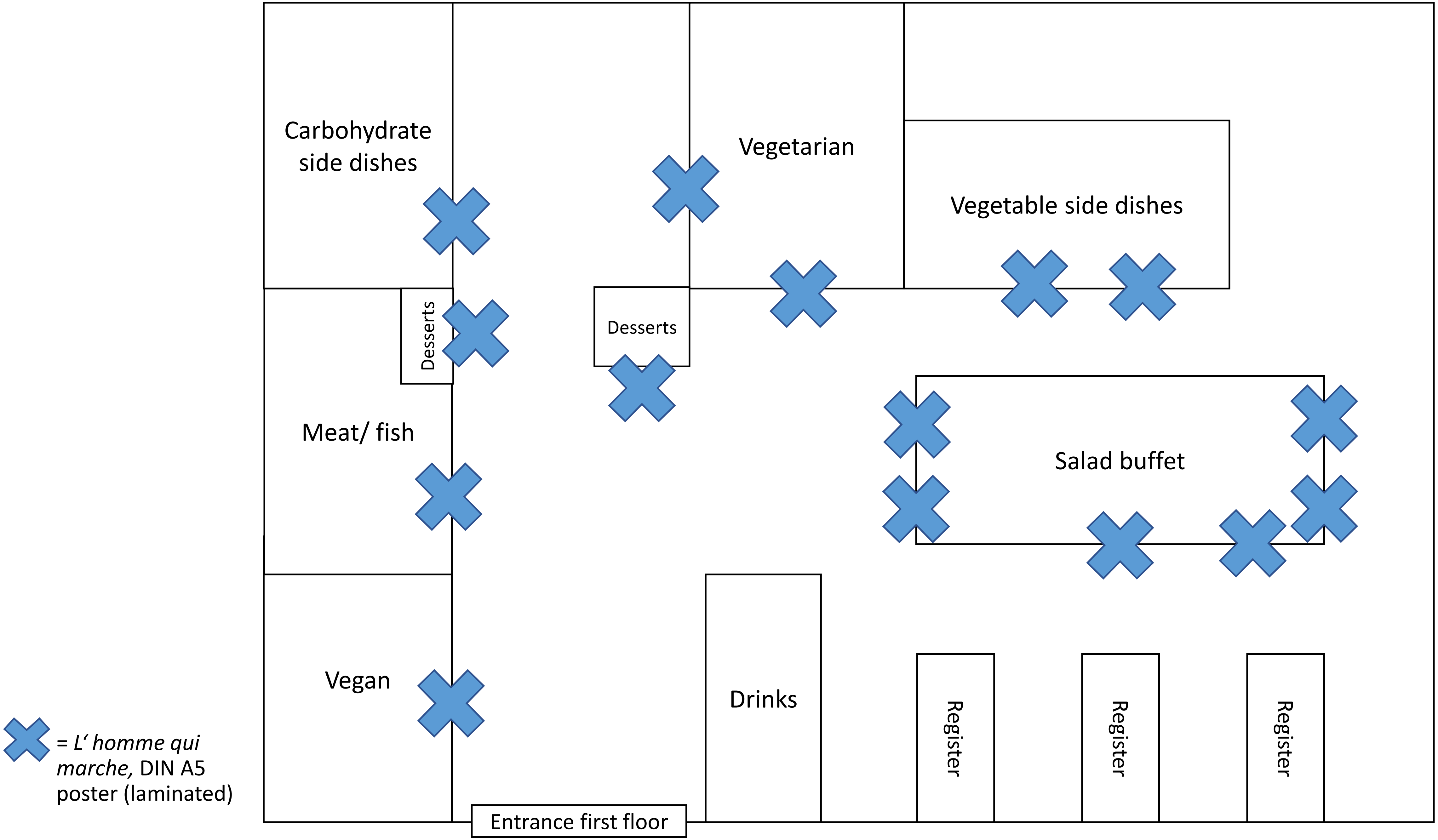

Supplement: Supplementary file 1 [file healthcare-11-01307-s001.zip › Supplementary Material S1_Layout of the cafeteria.pdf]
