# Supplementary material for: Effects of a Nudging Cue Targeting Food Choice in a University Cafeteria: A Field Study
Source: Healthcare (Basel). 2023 May 3;11(9):1307. doi: 10.3390/healthcare11091307 (PMC10178432; doi:10.3390/healthcare11091307)
Supplement: Supplementary file 1 [file healthcare-11-01307-s001.zip › Supplementary Material S2_ Questionnaire intervention week.pdf]

Liebe/r Teilnehmende/r,

diese Umfrage findet als Kooperation der Universität Maastricht, der Hochschule Bonn-Rhein-Sieg und der Mensa Campo statt. Sie ist Teil meiner Doktorarbeit zu den Effekten von Maßnahmen im Bereich Ernährung. Die Fragen behandeln z. B. die Gründe zur Menüwahl und die Akzeptanz von verschiedenen Maßnahmen zur Förderung von gesunder Ernährung.

Bei Fragen kannst du dich an Christine Kawa wenden:
Christine Kawa, M. Sc.
Hochschule Bonn-Rhein-Sieg
Von-Liebig-Str. 20
53359 Rheinbach
E-Mail: christine.kawa@h-brs.de

**INFORMIERTE TEILNAHME UND DATENSCHUTZ**

**Unversehrtheit und Rechte der Teilnehmer**
Durch die Teilnahme an dieser Studie entstehen keine seelischen oder körperliche Schäden. Deine Teilnahme ist freiwillig. Du hast jederzeit die Möglichkeit zu widerrufen. Durch Verweigerung oder Widerruf entstehen keine Nachteile. Ethische Begutachtung erfolgte durch das Ethics Review Committee Psychology and Neuroscience (ERCPN) der Maastricht University, Niederlande (Code ERCIC_368_26_06_2022).

**Datenschutz**
Die Daten werden ohne Rückschlüsse auf deine Person erhoben und ausgewertet (vollständig anonymisiert). Auf die anonymisierten Daten hat während und nach der Befragung nur Christine Kawa vollständigen Zugriff. Alle Angaben werden unter strenger Einhaltung der Datenschutzbestimmungen behandelt. Die Ergebnisse werden so veröffentlicht, dass eine Identifikation einzelner Personen nicht möglich ist. Die Daten werden nur so lange gespeichert, wie sie für die Durchführung der Studie benötigt werden (max. 2 Jahre). Bei Fragen wende dich gern per E-Mail an den Datenschutzbeauftragten der Hochschule Bonn-Rhein-Sieg: datenschutzbeauftragte@h-brs.de

Du hast als betroffene Person das Recht auf Berichtigung unrichtiger oder unvollständiger Daten (Artikel 16 SGVO). Zur Ausübung dieser Rechte kannst du dich jederzeit an Christine Kawa (Hochschule Bonn-Rhein-Sieg) wenden.

Es besteht das Recht zur Beschwerde bei der für die Hochschule Bonn-Rhein-Sieg zuständigen Aufsichtsbehörde:
Landesbeauftragte für Datenschutz und Informationsfreiheit Nordrhein-Westfalen
Kavalleriestr. 2-4, 40213 Düsseldorf, Tel.: 0211/38424-0, E-Mail: poststelle@ldi.nrw.de

**Ich habe die Informationen zur Studie und zur Verwendung meiner Daten gelesen. Ich hatte die Möglichkeit Fragen zu stellen und über meine Teilnahme nachzudenken. Ich bin 16 Jahre oder älter und stimme zu, an dieser Studie teilzunehmen.**

**Dies bestätige ich durch Ankreuzen:**

◯

Bitte umblättern.

**Menüwahl: VX#_________** **Datum: ________10.2022** **Uhrzeit: __________________**

1. **Hauptgericht:** ______________________________________________________________________
2. **Beilagen:** ______________________________________________________________________
3. **Dessert:** ______________________________________________________________________
4. **Bitte nenne die Gründe für deine Wahl**. (Mehrfachauswahl möglich)

◯ Geschmack
◯ Gewohnheit
◯ Hunger
◯ Gesundheit (weil es gesund ist)
◯ Einfachheit (weil es wenig Aufwand bedeutet)
◯ Genuss (weil ich mir etwas gönnen wollte)
◯ Tradition (weil ich damit aufgewachsen bin)
◯ Natürlichkeit (weil es naturbelassen ist)
◯ Gemeinschaft (weil es gesellig ist)
◯ Preis
◯ Präsentation (weil es mich anspricht)
◯ Gewichtskontrolle
◯ Gefühlsregulierung (um negative Gefühle zu kompensieren)
◯ Soziale Normen (weil es von mir erwartet wird)
◯ Soziales Image (weil es „in" ist)

5. **Wie hungrig warst du, als du dein Essen gewählt hast?**

◯ gar nicht hungrig ◯ kaum hungrig ◯ teils/ teils ◯ etwas hungrig ◯ sehr hungrig

Im Folgenden stellen wir dir eine Aussage zum Thema Akzeptanz von Maßnahmen im Bereich der Ernährung vor. Bitte gib an, inwiefern du diesen Aussagen zustimmst.

6. **Ich denke, es wäre akzeptabel, wenn die Mensa die folgenden Maßnahmen anwenden würde, um gesunde Ernährung zu bewerben:**

| | stimme nicht zu | stimme eher nicht zu | teils/ teils | stimme eher zu | stimme zu |
|---|---|---|---|---|---|
| Berühmte Personen als Informationsquelle | ◯ | ◯ | ◯ | ◯ | ◯ |
| Wettbewerb zum größten Gemüseverzehr | ◯ | ◯ | ◯ | ◯ | ◯ |
| Kampagnen mit abschreckenden Botschaften | ◯ | ◯ | ◯ | ◯ | ◯ |
| Informationen zum Gemüsekonsum Kommilitonen | ◯ | ◯ | ◯ | ◯ | ◯ |
| Grüner Salat als automatische Beilage (die auch abgewählt werden kann) | ◯ | ◯ | ◯ | ◯ | ◯ |
| Poster mit Tipps für einen höheren Gemüsekonsum | ◯ | ◯ | ◯ | ◯ | ◯ |
| Ansprache durch Mensa-Mitarbeiter, die nach zusätzlicher Gemüse-Auswahl fragen | ◯ | ◯ | ◯ | ◯ | ◯ |
| Ansprechendere Bezeichnung von Gerichten mit viel Gemüse | ◯ | ◯ | ◯ | ◯ | ◯ |
| Poster, auf denen sehr dünne künstlerische Skulpturen zu sehen sind | ◯ | ◯ | ◯ | ◯ | ◯ |

7. **Wie sehr hast du das Poster heute in der Mensa bewusst wahrgenommen?**

◯ gar nicht ◯ kaum ◯ teils/ teils ◯ etwas ◯ sehr

**Angaben zu deiner Person: Diese dienen nur dazu, die Stichprobe dieser Umfrage beschreiben zu können. Wenn du eine Frage nicht beantworten möchtest, überspringe diese Frage.**

8. **Zutreffendes bitte ankreuzen:** ◯ Ich sitze in einer **Gruppe** am Tisch ◯ Ich sitze **allein** am Tisch
9. **Alter**: __________Jahre
10. **Körpergröße**: ___________ cm (z.B. 163cm)
11. **Körpergewicht**: ____________ kg
12. **Geschlecht**: ◯ Männlich ◯ Weiblich ◯ Divers
13. **Status**: ◯ Studierender ◯ Unimitarbeiter ◯ Hochschulextern
14. **Wie viele Male hast du diese Woche schon in dieser Mensa gegessen:**

◯1 ◯2 ◯3 ◯4 ◯5

HERZLICHEN DANK für deine Teilnahme an dieser Umfrage!
